# Peptide mediated active targeting and intelligent particle size reduction-mediated enhanced penetrating of fabricated nanoparticles for triple-negative breast cancer treatment

**DOI:** 10.18632/oncotarget.5692

**Published:** 2015-10-12

**Authors:** Guanlian Hu, Xingli Chun, Yang Wang, Qin He, Huile Gao

**Affiliations:** ^1^ Key Laboratory of Drug Targeting and Drug Delivery Systems, West China School of Pharmacy, Sichuan University, Chengdu 610041, China; ^2^ State Key Laboratory of Molecular Engineering of Polymers (Fudan University), Shanghai 200433, China

**Keywords:** active targeting, particle size reduction, tumor penetration, triple-negative breast cancer, gelatin nanoparticles

## Abstract

Triple-negative breast cancer (TNBC) is one of the most invasively malignant human cancers and its incidence increases year by year. Effective therapeutics against them needs to be developed urgently. In this study, a kind of angiopep-2 modified and intelligently particle size-reducible NPs, Angio-DOX-DGL-GNP, was designed for accomplishing both high accumulation and deep penetration within tumor tissues. On one hand, for improving the cancerous targeting efficiency of NPs, angiopep-2 was anchored on the surface of NPs to facilitate their accumulation via binding with low density lipoprotein-receptor related protein (LRP) overexpressed on TNBC. On the other hand, for achieving high tumor retention and increasing tumor penetration, an intelligently particle size-reducible NPs were constructed through fabricating gelatin NPs (GNP) with doxorubicin (DOX) loaded dendrigraft poly-lysine (DGL). *In vitro* cellular uptake and *ex-vivo* imaging proved the tumor targeting effect of Angio-DOX-DGL-GNP. Additionally, the degradation of large-sized Angio-DOX-DGL-GNP by matrix metalloproteinase-2 (MMP-2) led to the size reduction from 185.7 nm to 55.6 nm. More importantly, the penetration ability of Angio-DOX-DGL-GNP after incubation with MMP-2 was dominantly enhanced in tumor spheroids. Due to a combinational effect of active targeting and deep tumor penetration, the tumor growth inhibition rate of Angio-DOX-DGL-GNP was 74.1% in a 4T1 breast cancer bearing mouse model, which was significantly higher than other groups. Taken together, we successfully demonstrated a promising and effective nanoplatform for TNBC treatment.

## INTRODUCTION

Triple-negative breast cancer (TNBC), which does not express or express low levels of estrogen receptor, progesterone receptor and HER2/neu, is one of the most invasively malignant human cancers and its incidence increases year by year [1, 2]. Currently, there only exist few standard therapies for TNBC [3]. The development of nanoparticles (NPs), as tools of nanomedicine, has made significant advances towards TNBC treatment [4]. Unfortunately, the therapeutic efficiency of NPs is usually unsatisfied [5–7]. Firstly, the targeting ability of conventional NPs is too low to effectively accumulate around tumor sites, reducing the concentration of chemical drugs delivered by NPs and compromising the therapeutic effect. Secondly, after accumulating around tumors, NPs faced vastly diffusional hinderance due to the presence of the compressed intratumoral blood and lymphatic vessels and the dense collagen-rich extracellular matrix (ECM). Therefore, they were always unable to access into the nonvascularized and anoxic regions within deep tumor parenchyma, and the cancerous cells of core area are still aggressively survived, resulting in chemotherapeutic bland and latent crisis for tumor metastasis and regeneration [8, 9]. Based on these considerations, the development of novel NPs for TNBC is still urgently needed and the above two issues should be addressed.

For improving the tumor targeting efficiency of NPs, a variety of ligands are modified on the surface of NPs in favor to actively tumorous accumulation [10–12]. Considering the overexpression of LRP1 on TNBC cells, the corresponding peptide, angiopep-2 was employed to decorate NPs to endow our NPs with active TNBC targeting ability [13]. Furthermore, size-alterable NPs are constructed for overcoming the diffusional hinderance within tumors. Compared with small-sized NPs, NPs with larger size (100–200 nm) usually possessed higher tumor accumulation abilities [14, 15], but their diffusional abilities within tumor were severely impeded by the dense ECM and high tumor interstitial pressure, thus yielding a heterogeneous distribution in solid tumors [16, 17]. In contrast, small-sized NPs displayed superior permeability within tumor tissues but largely restricted by fast clearance *in vivo* [18–21]. Thus size-reducible NPs were developed for addressing the low tumor accumulation and limited tumor penetration. Taking together, angiopep-2 modified and intelligently size-reducible NPs were developed to effectively deliver drugs to TNBC.

**Figure 1:**
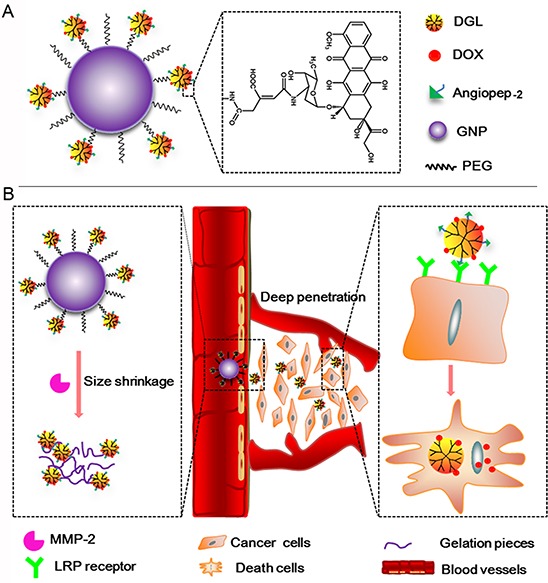
A. The schematic illustration of Angio-DOX-DGL-GNP, the linker between DOX and DGL was acid-sensitive cis-aconitic anhydride bond. B. The schematic illustration of the delivery of Angio-DOX-DGL-GNP in breast cancer cells Large-sized Angio-DOX-DGL-GNP accumulated around the perivascular sites through active and passive targeting and then underwent large-to-small size transition by MMP-2 overexpressed in TNBC for deep tumor penetration; LRP receptor mediated cellular uptake of Angio-DOX-DGL-PEG and cell apoptosis induced by the released DOX.

In our study, Angio-DOX-DGL-GNP with core-shell nanostructure, were designed to integrate the active tumor targeting and size-shrinkable property. As shown in Figure 1, the core was composed of gelatin NPs (GNP) degraded by MMP-2, while the shell was made up of DGL linked with DOX and angiopep-2. During blood circulation, Angio-DOX-DGL-GNP could effectively accumulate around in tumor sites through passive and active tumor targeting [22, 23]. Then the core of Angio-DOX-DGL-GNP was dissembled when exposed to MMP-2 overexpressed in TNBC [24–26] and released the small-sized Angio-DOX-DGL-PEG, facilitating the delivery of NPs to the core area to kill more viable cells [24, 27]. Specifically, in consideration of the uniformly small size (4 ~ 7 nm) and structural modifications for specific biomedical applications, DGL was employed as the small-sized carriers linked with angiopep-2 [28–30]. Moreover, with the aim to examine the ability of Angio-DOX-DGL-GNP's targeting effect and therapeutic effect, DOX was attached to DGL via a pH-sensitive cis-aconitic anhydride bond [31, 32]. In deed, our *in vitro* and *in vivo* results indicated that Angio-DOX-DGL-GNP possessed notable tumor accumulation and penetration abilities, thus maximizing antitumor effect.

## RESULTS

### Synthesis and characterization of Angio-DOX-DGL-GNP

DOX was conjugated to the residual primary amino groups of Angio-PEG-DGL by cis-aconityl bond. Infrared spectrometry exhibited the absorption band of the primary amino of DOX was 3328 cm^−1^ and 3523 cm^−1^, while cis-aconitic anhydride-doxorubicin (CAD) was in 3423 cm^−1^ consistent with the absorption band of the secondary amino groups, indicating the formation of amide bond and successive synthesis of CAD (Figure S1A and S1B). In NMR spectra of Angio-PEG-DGL, the solvent peak of D_2_O was found at 4.65 ppm. The repeat units (-O-CH_2_-CH_2_-O-) of PEG presented as a sharp peak at 3.4–3.6 ppm (Figure S1C). The methylene protons of branching units of DGL had double peaks between 1 ppm and 2 ppm. The peaks between 6.7 ppm and 7.3 ppm proved the existence of angiopep-2. Finally, the activated carboxyl groups of CAD were grafted on the amino groups of Angio-DGL-PEG via amide linkage to obtain the final product: Angio-DOX-DGL-PEG.

The particle size of Angio-DOX-DGL-PEG and Angio-DOX-DGL-GNP were 35.1 ± 1.7 nm and 185.7 ± 3.2 nm respectively with a narrow distribution. The drug content of Angio-DOX-DGL-PEG and Angio-DOX-DGL-GNP were 10.32 ± 0.3% and 4.82 ± 0.2%, respectively. As determined by MTT (Figure S2), the constructed DGL-GNP was safer than DGL, suggesting the carrier was biocompatible.

### *In vitro* release of DOX

The *in vitro* release of DOX from Angio-DOX-DGL-GNP was performed at pH 7.4, 6.0, 5.0 and 3.0 (Figure S3). The drug release rate obviously increased as reduction of pH value. Little amount of DOX released at pH 7.4 and the cumulative release amount was less than10% after 24 h, suggesting the stability of cis-aconitic anhydride bond under neutral pH. When the pH decreased to 5.0, the 24 h cumulative release amount was nearly 85%, indicating the acidic sensitivity of cis-aconitic anhydride bond.

### Degradation of GNPs triggered by MMP-2

**Figure 2:**
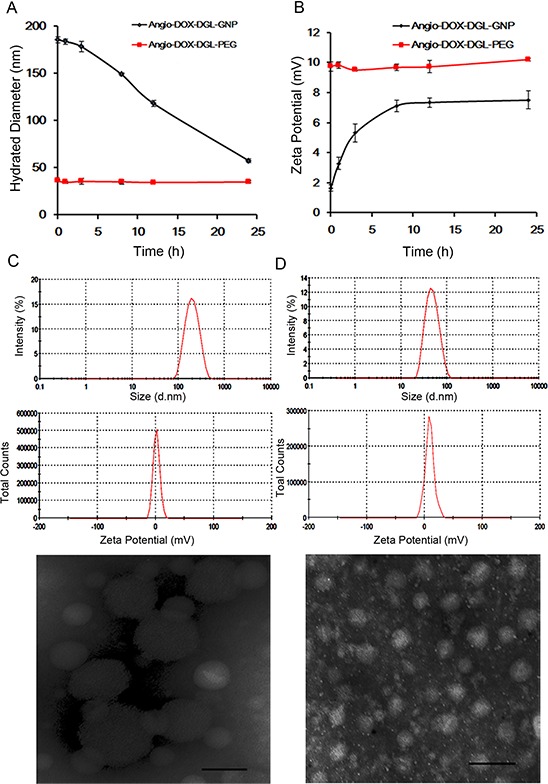
**A. Hydrated Diameter of Angio-DOX-DGL-GNP and Angio-DOX-DGL-PEG incubated with MMP-2 during 24 h. B.** Zeta Potential of Angio-DOX-DGL-GNP and Angio-DOX-DGL-PEG incubated with MMP-2 during 24 h. **C.** DLS data (Size distribution of Zeta potential) and TEM images of Angio-DOX-DGL-GNP before incubation with MMP-2, bar represents 100 nm. **D.** DLS data (Size distribution of Zeta potential) and TEM images of Angio-DOX-DGL-GNP after incubation with MMP-2 for 24 h, bar represents 100 nm.

Particle size of Angio-DOX-DGL-GNP notably decreased from 185.7 nm to 55.6 nm in the presence of MMP-2, which was attributed to the degradation of gelatin in Angio-DOX-DGL-GNP (Figure 2). Moreover, the zeta potential of Angio-DOX-DGL-GNP increased from +1.65 mV to +7.48 mV, which may facilitate the cellular uptake in tumor sites. However, the diameter of Angio-DOX-DGL-PEG remained stable and the transmission electron microscope (TEM) image also proved the same shrinkable properties. More importantly, the degradation of the GNP core by MMP-2 facilitated the release of small-sized Angio-DOX-DGL-PEG at tumor sites, which can largely decrease the diffusional hinderance. All the results suggested Angio-DOX-DGL-GNP could accomplish large-to-small shrinkage in response to MMP-2, thereby penetrating into the core area in tumor sites.

### *In vitro* penetration efficiency of Angio-DOX-DGL-GNP2

**Figure 3:**
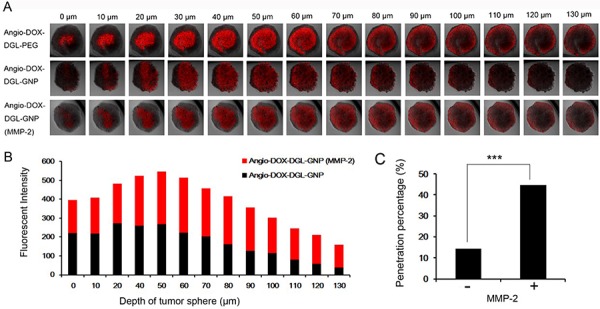
**A. Fluorescent images of 4T1 MCTs after incubation with Angio-DOX-DGL-GNP and Angio-DOX-DGL-GNP (MMP-2) for 24 h. B.** Semi-quantitative intensity of inner region at different sections of 4T1 tumor spheroids. **C.** Penetration percentage of Angio-DOX-DGL-GNP at 130 μm.

4T1 cells, which are extremely aggressive, highly tumorigenic and metastatic, is selected as a model for TNBC [2, 33, 34]. The multicellular tumor spheroids (MCTs) were employed to evaluate the penetration efficiency of Angio-DOX-DGL-GNP after degradation by MMP-2 because the MCTs' environment was similar to *in vivo* tumors, such as poor drug penetration, free of microvessel, changed protein expression and activity and gradients of oxygen tension and nutrients [35, 36]. The distribution of different formulations in superficial sections of 4T1 MCTs was observed. MCTs treated with Angio-DOX-DGL-PEG exhibited higher intensity in all slices than that of Angio-DOX-DGL-GNP, implying that smaller-sized particles possessed better penetrating efficiency. Moreover, Angio-DOX-DGL-GNP (with MMP-2) displayed the similar penetration ability as small-sized Angio-DOX-DGL-PEG, which was largely attributing to the size shrinkable properties of Angio-DOX-DGL-GNP in presence of MMP-2 (Figure 3A). By comparison, Angio-DOX-DGL-GNP was located at the edge of MCTs, while the fluorescence of Angio-DOX-DGL-GNP pre-incubated with MMP-2 distributed more extensively and penetrated much deeper in the distance of 100 μm than that of Angio-DOX-DGL-GNP groups (Figure 3B). For better evaluated the permeability of NPs, the penetrating percentage (fluorescent intensity of inner region to edge region) at 130 μm depth was introduced to determine the penetrating efficiency (Figure 3C). After pre-treatment with MMP-2, the penetrating efficiency of Angio-DOX-DGL-GNP in MCTs was apparently improved. The results confirmed that Angio-DOX-DGL-GNP possessed better penetration ability after incubation with MMP-2 and the reduction of particle size was beneficial for enhancing penetrating efficiency through the tumor spheroids experiments.

### *In vitro* cellular uptake study

**Figure 4:**
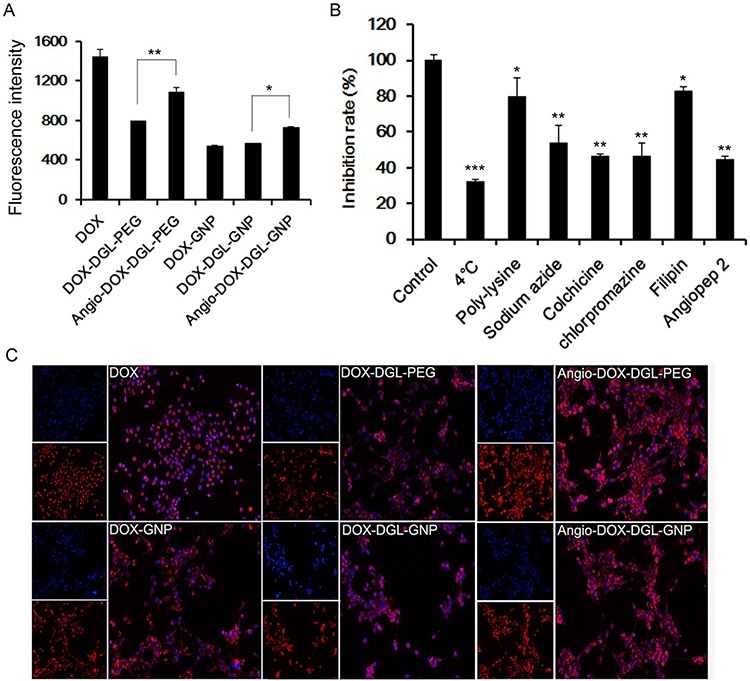
**A. Cellular uptake of 4T1 cells after incubation with different formulations for 2 h. Data represent the mean ± SD (*n* = 3). B.** The endocytosis inhibition assay of Angio-DOX-DGL-GNP on 4T1 cells. The inhibition rate (%) is expressed as the ratios of the cellular uptake in the presence of various inhibitors to the uptake in absence of inhibitor. **C.** Images of 4T1 cells treated with DOX, DOX-DGL-PEG, Angio-DOX-DGL-PEG, DOX-GNP, DOX-DGL-GNP and Angio-DOX-DGL-GNP.

In order to validate the enhanced cellular uptake of Angio-DOX-DGL-GNP, 4T1 cells were incubated with these NPs for 2 h. The fluorescent intensity of Angio-DOX-DGL-GNP was significantly stronger than that of DOX-DGL-GNP, which confirmed that Angio-DOX-DGL-GNP possessed stronger ability of entering into tumor cells (Figure 4A and 4C).

### *In vitro* cellular uptake mechanism

Different inhibitors were utilized to conduct the endocytosis inhibition assay. 4°C and sodium azide were employed to deplete cellular ATP in order to explore the impact of energy on the cellular uptake. Colchicine, chlorpromazine and filipin were chosen to block macropinocytosis-mediated, clathrin-mediated and caveolin-mediated endocytosis respectively and poly-lysine was chosen as positive charge inhibitor [39, 40]. Ligands on the surface of NPs also correlate with the internalization of NPs. Our data exhibited that various mechanisms were involved in the uptake process. The cellular uptake was remarkably reduced under 4°C and sodium azide, implying that the endocytosis of Angio-DOX-DGL-GNP was energy-correlated. Moreover, the rate of uptake was also significantly inhibited by colchicine, chlorpromazine and angiopep-2, indicating that macropinocytosis, clathrin- and receptor-mediated endocytosis were involved in the cellular uptake of Angio-DOX-DGL-GNP.

### *In vitro* cellular apoptosis assay

**Figure 5:**
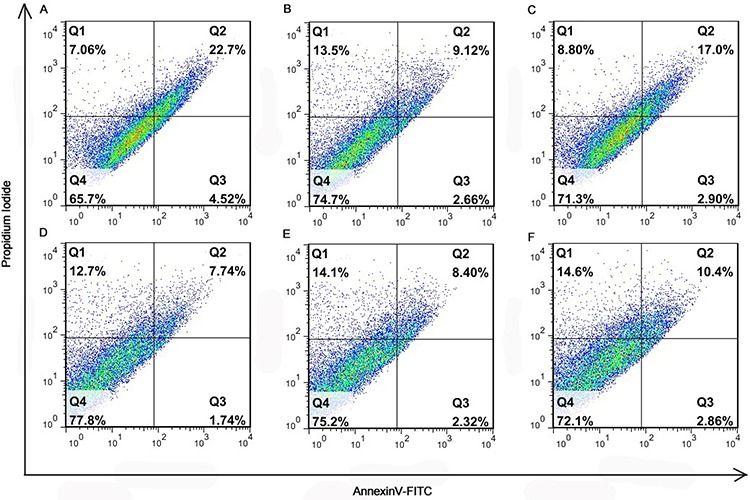
The diagrams of the apoptosis assay of different formulations **A.** DOX; **B.** DOX-DGL-PEG; **C.** Angio-DOX-DGL-PEG; **D.** DOX-GNP; **E.** DOX-DGL-GNP; **F.** Angio-DOX-DGL-GNP.

As demonstrated in Figure 5, the apoptosis of 4T1 cells induced by Angio-DOX-DGL-GNP was higher than that by DOX-DGL-GNP, suggesting angiopep-2 modification could enhance the antitumor effect. However, the apoptosis percentage of Angio-DOX-DGL-GNP was not higher than that of Angio-DOX-DGL-PEG, which was because the particles directly contacted with cells and this study could not reflect the difference between particles with different sizes.

### *In vivo* tumor distribution and penetration

**Figure 6:**
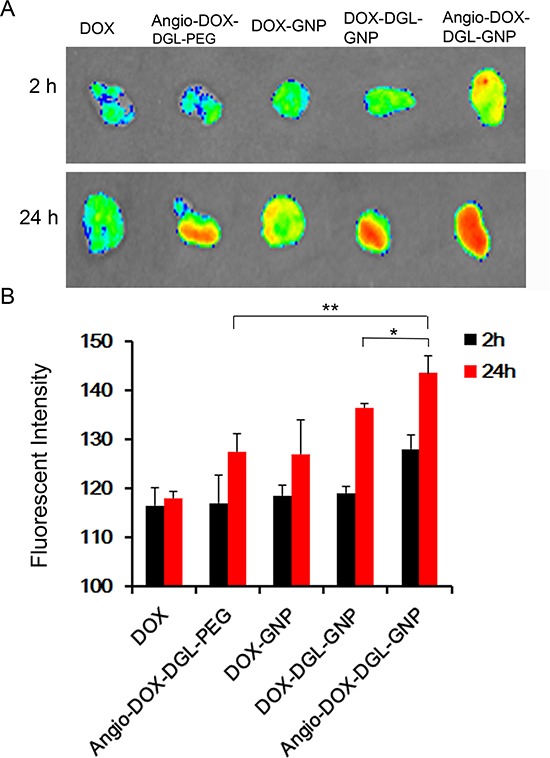
**A.** *Ex vivo* fluorescence imaging of the tumor and normal tissues of 4T1 tumor-bearing BALB/C mice after 2 h or 24 h post-injection of different DOX formulations. **B.** Semi- quantitative analysis of fluorescent intensity of tumor sites.

**Figure 7:**
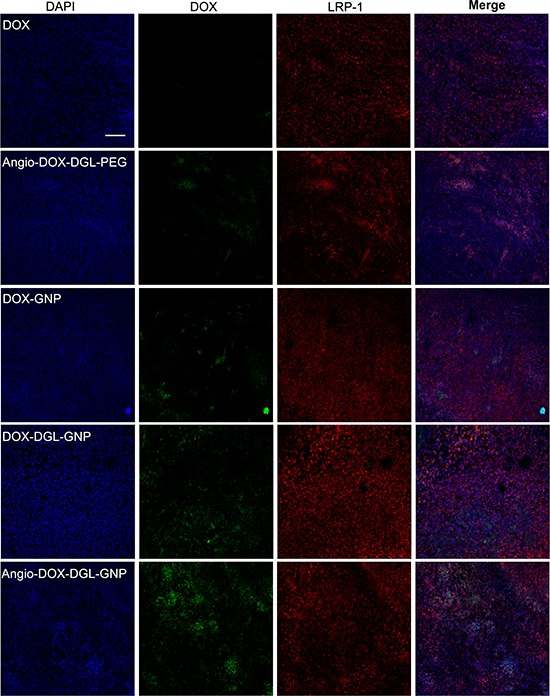
*In vivo* distribution of DOX, Angio-DOX-DGL-PEG, DOX-GNP, DOX-DGL-GNP and Angio-DOX-DGL-GNP in breast cancer Blue represents DAPI; Green represents the fluorescence of DOX; Red represents LRP-1. Bar represents 100 μm.

**Figure 8:**
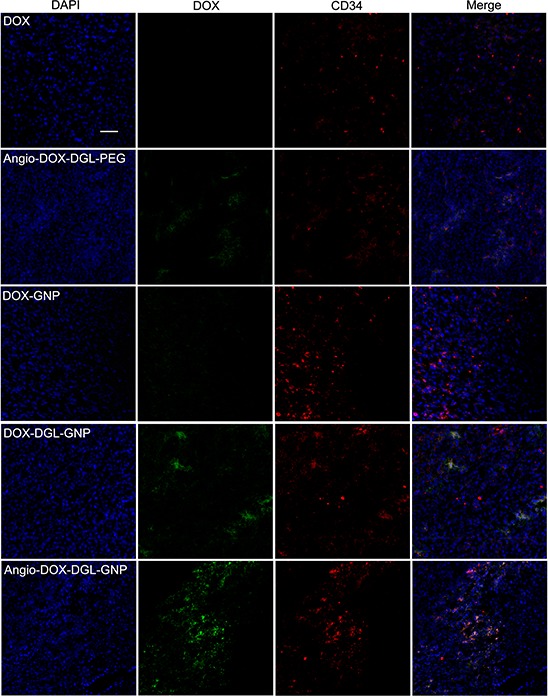
*In vivo* tumor distribution of DOX, Angio-DOX-DGL-PEG, DOX-GNP, DOX-DGL-GNP and Angio-DOX-DGL-GNP Blue represents DAPI, green represents the fluorescence of DOX, red represents CD34 labeled blood vessels and bar represents 50 μm.

To test the biodistribution and tumor targeting of Angio-DOX-DGL-GNP *in vivo*, a murine 4T1 xenograft model of TNBC was established and a real-time fluorescence imaging technique was applied. At 24 h post-injection, the large-sized Angio-DOX-DGL-GNP exhibited a stronger fluorescent signal at tumor site than small-sized Angio-DOX-DGL-PEG (Figure 6, S4), which attributed to the enhanced tumor retention effect. More importantly, the fluorescent intensity of Angio-DOX-DGL-GNP in tumor region was higher compared with that of other groups, validating the significant tumor targeting effect of angiopep-2 (Figure 7). Accordingly, it was demonstrated that Angio-DOX-DGL-GNP had a high accumulation in tumor sites as a result of a combination of the passive and active targeting mechanisms. To further visualize the distribution of NPs, the tumor slices was stained with CD34 and LRP-1 (Red) and the distribution of DOX (green) was captured (Figure 7–8). The fluorescent intensity of Angio-DOX-DGL-GNP was remarkably higher than other groups, which benefited from better tumor targeting and penetration effect. Although many of Angio-DOX-DGL-GNP colocalized with microvessel, there were also fluorescence distributed in the area free of vessel (Figure 8), suggesting the Angio-DOX-DGL-GNP could penetrate deeper in tumor than other groups.

### *In vivo* therapy studies

**Figure 9:**
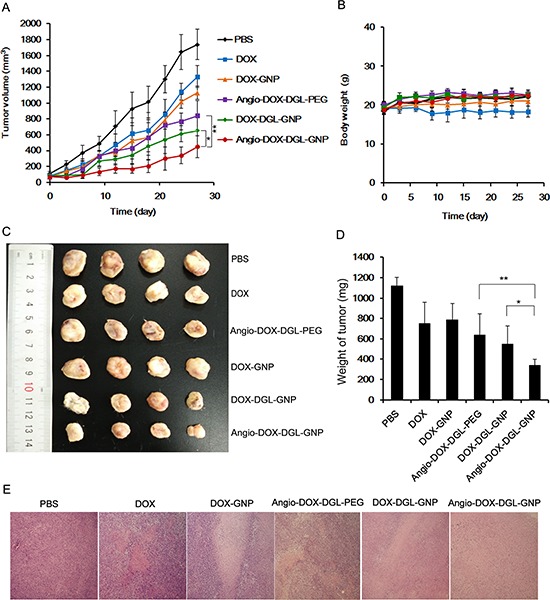
*In vivo* tumor growth inhibition experiment **A.** The 4T1 tumor growth curves after intravenous injection of different formulations of DOX. **B.** The body weight of mice after administration of different formulations of DOX and PBS up to day 27 (*n* = 6). **C.** The images of tumors of mice obtained from sacrificed mice at the end of this experiment. **D.** The weight of tumor after 27 days treatment (*n* = 6). **E.** Histological images of tumors sections stained by H&E (tumors: × 100).

**Figure 10:**
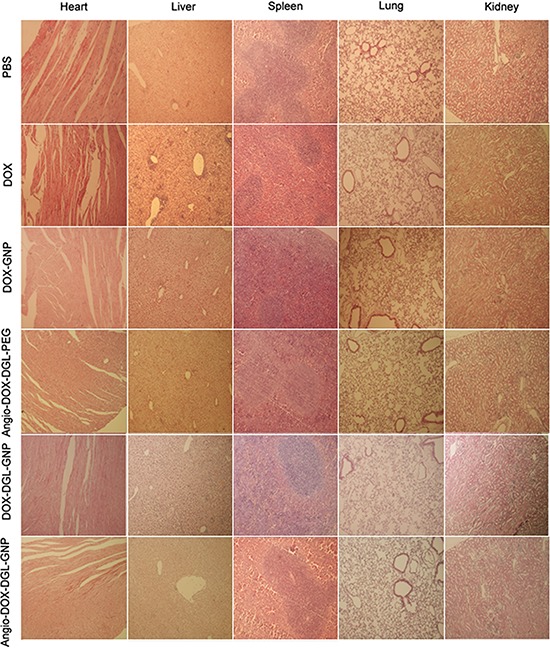
Histological analysis for different organs of 4T1 bearing mice administrated PBS, DOX, Angio-DOX-DGL-PEG, DOX-GNP, DOX-DGL-GNP and Angio-DOX-DGL-GNP (all tissues: × 100)

To demonstrate the feasibility of Angio-DOX-DGL-GNP for cancer treatment *in vivo*, the therapeutic efficacy of Angio-DOX-DGL-GNP compared with several other NPs was evaluated in 4T1 tumor bearing mice. The rate of tumor growth was suppressed to different degree after intravenous administration of various formulations compared with the control group (Figure 9). Angio-DOX-DGL-GNP exerted a noticeably higher effect on tumor inhibition compared with non-targeted NP (DOX-GNP, DOX-DGL-GNP) and small-sized Angio-DOX-DGL-PEG, which mainly ascribed to better tumor retention effect combined with the active target ability provided by angiopep-2. More importantly, Angio-DOX-DGL-GNP cannot only accumulate more efficiently at tumor sites via active targeting but also penetrate deeper within the tumor. During the entire treatment period, no noticeable alteration of mice body weight was observed in all groups except DOX group, which was due to the high toxicity of free DOX. Moreover, the images of tumor slices using hematoxylin and eosin (H&E) staining presented a massive cancer cell remission after administration of Angio-DOX-DGL-GNP (Figure 9E), suggesting Angio-DOX-DGL-GNP could induce more apoptosis tumor cells. The histologic images of main tissues stained by H&E showed that DOX induced the necrosis of heart with acute inflammatory cells infiltration (Figure 10). In contrast, no noticeable changes of Angio-DOX-DGL-GNP groups were visualized in comparison with control group, indicating the application of them *in vivo* was safe. Taking together, the Angio-DOX-DGL-GNP significantly enhanced tumor targeting efficacy and possessed better antitumor efficacy and low side effect.

## DISCUSSIONS

Many factors *in vivo* constitute challenging hurdles for TNBC treatment such as high interstitial fluid pressure, dense stromal tissue, complex interaction with fibroblasts and tumor cells, thus conventional NPs were not homogeneously distributed in tumor sites and just achieved compromising therapeutic effects. Many strategies were addressed to eradicating the bland area of chemotherapy such as size-reduction, depletion of ECM and pulsed high intensity focused ultrasound [16, 27, 37, 38]. Among them, size-reducible NPs hold most attention for its incomparable advantages such as high accumulation and superior tumor penetration [37, 39]. For example, Daniel S. Kohane reported a photo-triggered NPs undergoing large-to-small size shrinkage from 150 nm to 40 nm [27]. In another study, Fukumura designed a QDs-GNP with shrinkable size triggered by MMP-2, thus releasing small-sized QDs for deep tumor penetration [24, 40].

In our study, significantly higher cellular uptake of Angio-DOX-DGL-GNP was observed in 4T1 cells indicating that angiopep-2 modifying facilitated the cellular uptake of Angio-DOX-DGL-GNP. Furthermore, size shrinkage NPs were designed to overcome the hinderance of tumor region, thus maximizing the antitumor effect. In our study the degradability of GNP core was evaluated by monitoring the size change of nanocarrier after incubation with MMP-2. The results showed that the size of Angio-DOX-DGL-GNP could reduce from 185.7 nm to 55.6 nm, indicating GNP could be degraded into small fragments by MMP-2 as previous study showed [41, 42]. *In vivo* imaging experiment also demonstrated that Angio-DOX-DGL-GNPs accumulated in tumor tissues more efficiently via EPR effect and active targeting in 4T1 tumor bearing mice. Our study expected that after the GNP core were degraded by MMP-2, the released small-sized Angio-DOX-DGL-PEG penetrated into the regions away from the tumor blood vessels to kill more cancer cells. However, the penetration ability of Angio-DOX-DGL-GNP *in vivo* wasn't satisfied which may due to the sensitivity to MMP-2 degradation, but in the evaluation of anti-TNBC effect in 4T1 grafted model, Angio-DOX-DGL-GNP exhibited the highest tumor growth inhibition rate than other groups, implying that Angio-DOX-DGL-GNP accomplished our targets to some extent. An ideal enzyme-responsive multistage NPs should been rapidly degraded into small-sized NPs after they accumulated in tumor sites, therefore small-sized NPs penetrated into the core of tumor sites and released the anticancer drug, leading to killing more tumor cells. In fact, when the NPs reached the tumor sites by EPR effect, the multistage NPs were degraded into small NPs in accompany with penetration. The smaller the particle size of NPs shrank, the deeper the NPs penetrated. Certainly, further research was also needed for us to increase the responsiveness of our NPs in the future. More importantly, in the aspect of the *in vivo* application of safety of Angio-DOX-DGL-GNP, no significant weight change and hematoxylin and eosin staining results proved that these nanocarrier were of biological safety, which was corresponded with other researches [43, 44].

## MATERIALS AND METHODS

### Materials

Doxorubicin hydrochloride was purchased from Beijing Huafenglianbo Technology Co. Ltd. (Beijing, China). Aconitic anhydride (CA) was purchased from Alfa Aesar Chemical Co. Ltd. (Tianjin, China). DGL-G3 dendrimer was purchased from Colcom (Montpellier Cedex, France). Gelatin type A was purchased from MP Biomedicals Co. Ltd. (California, USA). Glutaraldehyde solution (Grade II, 25%) was purchased from Beijing Solarbio Technology Co. Ltd. (Beijing, China). Angiopep-2 was purchased from Qiangyao Biotechnology Co. Ltd. (Shanghai, China). Amino-PEG_5000_-succinimidyl carbonate, α-mPEG_5000_-ω-amino and dicarboxyl-PEG_5000_ were purchased from Seebio Biotechnology Co. Ltd. (Shanghai, China). 1-Ethyl-3-(3-dimethylaminopropyl) carbodiimide (EDC) and *N*-hydroxy-succinimide (NHS) were purchased from Keddia Reagent Co. Ltd. (Chengdu, China). 6-Diamidino-2-pheylindole (DAPI) was obtained from Beyotime Insitute Biotechnology (Haimen, China). Anti-CD34 antibody was purchased from Abcam (Hong Kong). Alexa Fluor 594-conjugated AffiniPure donkey anti-rabbit was purchased from Jackson Immuno Research Laboratories Inc (West Grove, USA). Rb mAb to LRP-1 (EPR 3724) was purchased from Abscam (Hong Kong). Cy3-conjugated Affinipure Goat Anti-Rabbit IG (H+L) was purchased from Jackson ImmunoRearch Laboratories, Inc.(West Grove, USA). MMP-2 protein (rat) was purchased from Abcam Ltd. (Hong Kong, China). Purified rabbit mAb to CD34 was purchased from Abcam Ltd. (Hong Kong, China). RPMI1640, 3-(4, 5-dimethylthialzolayl)-2, 5-diphenyltetrazolium bromide (MTT) was purchased from Baoxin Biotechnology Ltd. (Chengdu, China). Mouse mammary breast tumor cell line (4T1) was purchased from Shanghai Institute of Cell Biology (Shanghai, China). All other reagents and solvents were of analytical or HPLC grade and were used without further purification.

### Synthesis of Angio-DOX-DGL-PEG

The conjugation of DOX to DGL via cis-aconityl bond was performed as previously reported literature [32, 45]. Briefly, cis-aconitic anhydride (CA) (54 mg) dissolved in 1 mL of 1, 4-dioxane was added dropwise to the doxorubicin solution (30 mg, 51.7 μmol) with continuous stirring in dark at room temperature and the pH value was controlled in the range of 8.4–8.7 by NaOH. Then a great amount of heavy precipitate was produced by slowly adding in HCl (1 M) in ice bath and distributed in 25 mL of ethyl acetate for 4 times. After the organic solvent was removed by the rotary evaporator, the structure of CAD was determined by Infrared Spectrometer (VECTOR22) for FT-IR analysis. Secondly, DGL was reacted with the activated dicarboxyl-PEG_5000_ at the ratio 1: 8 (molar ratio) in pH 8.0 PBS for 2 h in the presence of EDC and NHS followed by ultrafiltration by using a 10KDa molecular weight cutoff membrane to remove the small molecules. Angiopep-2 was added to the purified mixture at the ratio of 1: 8 (DGL to peptide, molar ratio) for 24 h in the presence of EDC and NHS, finally the reaction mixture was dialyzed against deionized water twice for 12 h. Next the purified reaction was freeze-dried to obtain a white power and analyzed in a 400 MHz spectrometer. At last, a 10-fold molar excess of EDC and NHS was added to CAD in 2 mL of PBS and reacted under the dark condition for 0.5 h. Then the solutions (CAD: Angio-PEG-DGL = 48: 1, molar ratio) were mixed and reacted for another 12 h. At last, the unreacted CAD and other small molecules were removed by ultrafiltration through a 10KDa molecular weight cutoff membrane.

### Preparation of GNPs

GNPs were fabricated by a two-step desolvation method as previous reports with minor modification [46, 47]. Briefly, 625 mg of gelatin type A was dissolved in 12.5 mL of deionized water (DI) at 40°C, then 12.5 mL of acetone was added to the gelatin solution under low-speed stirring. Stop stirring exactly 1 min, the supernatant was thrown away and 8 mL of DI was added to the solution. Then acetone was added to the solution (pH 2.7–3.0) until a sustained faint turbidity was visualized, followed by addition of 60 μL of 25% glutaraldehyde solution diluted in 1 mL of acetone to harden the NPs. Finally, the solution was stirred at 40°C and 600 rpm for 7 h and the acetone was removed. 0.2 mL of 1 M glycine solution was added to terminate the cross linking. Finally the GNPs were purified by passage through a Sephadex G-50 column.

### Preparation and drug content of Angio-DOX-DGL-GNP

1 mL of GNP solution (20 ~ 25 mg) was activated by an additional solution of EDC (0.8 mg) and sulfo-NHS (0.8 mg) for 30 min, then COOH-PEG_5000_-NH_2_ (20 mg, × 2 μmol) was added to the activated GNP solution to obtain GNP-PEG_5000_-COOH for 2 h. Next the carboxyl groups of GNP-PEG_5000_-COOH were activated by an additional solution of EDC (2 mg) and sulfo-NHS (2 mg) in 50 μL of DI under pH 6.0 for 30 min. After the pH value of the mixture was adjusted to 8.0 again, Angio-DOX-DGL-PEG (equivalent to 0.5 mg DOX) was added and stirring for another 8 h to obtain Angio-DOX-DGL-GNP. Finally, the solutions were purified by ultrafiltration through a 100KDa molecular weight cutoff membrane at 4500 g for 30 min.

Drug content was quantified by hydrolyzing the glycosidic bond between the doxorubicinone and amino sugar at acidic condition and determining the released doxorubicinone. Different formulations (0.1 mL) was mixed with 2.5 M HCl (0.8 mL) and methanol (1 mL), and the mixture was incubated at 50°C for 1.5 h. The generated doxorubicinone was analyzed by HPLC (WelchromR C18 column (4.6 × 250 mm,5 mm particle size, Agilent 1200 series), 0.01 M KH_2_PO_4_ : acetonitrile : acetic acid = 45 : 55 : 0.27 (v/v/v), 1.0 mL/min, 30°C, 490 nm). A standard curve of doxorubicinone was generated by hydrolyzing free DOX at the same condition, then the amount of DOX conjugation were obtained and the drug loading efficiency were finally calculated.

### *In vitro* release

As the linker between DOX and DGL was a pH-sensitive linker, cis-aconitic anhydride bond, *in vitro* release profiles of Angio-DOX-DGL-GNP (equivalent to 0.1 mg DOX) were performed in 40 mL of PBS (pH 7.4, 6.0, 5.0 and 3.0) medium at 37°C and 50 rpm. The samples were sealed in a dialysis bag (MW 3500). At predetermined intervals, 1 mL of released media was taken away and the corresponding fresh medium was added. The amount of released DOX was measured by fluorescence spectrophotometer. Each drug release test was performed thrice. Each drug release test was performed thrice.

### Degradation of GNP triggered by MMP-2 *in vitro*

MMPs, especially MMP-2 and MMP-9, are associated with the invasion, progression angiogenesis and metastasis of many human cancers. The expression levels of MMPs were found to be relatively high in breast cancer, liver cancer, liver cancer, lung cancer and ovarian cancer, whereas they are minimally expressed in healthy tissue [48–51]. To investigate the enzyme-sensitivity of Angio-DOX-DGL-GNP, 0.5 mL of Angio-DOX-DGL-GNP (0.4 mg) was incubated with 0.5 mL of MMP-2 in HEPES (460 ng). At prearranged time intervals, the particle size and zeta potential of the NPs were immediately determined by a dynamic light scattering detector (Nano-ZS, Malvern, UK). The morphology of Angio-DOX-DGL-GNP before or after MMP-2 incubation was observed via TEM (JEM-100CX, JEOL, Japan).

### Penetration assay using MCTs

4T1 cells were seeded into 96-well plates pre-coated with sterile agarose solution (2%, m/v) at a density of 8 × 10^3^ cells per well. Subsequently, the MCTs were monitored with optical microscope in order to assure that they form intact spheres and ready to use after 3 days. Then MCTs were selected with uniform size and then incubated with different formulations for 12 h. Subsequently, the MCTs were rinsed thrice with cold PBS followed by fixation of 4% paraformaldehyde for 30 min and placed in 96-well plates for confocal microscopy.

### *In vitro* cellular uptake study

The cellular uptake of different formulations was evaluated by fluorescent microscope, 4T1 were seeded at a density of 5 × 10^4^ cells/well on 6-well plates containing square coverships for 24 h. After achieving 70%–80% confluence, various samples (12.5 μg/mL, DOX equal) was added to each well and incubated for 2 h. Then the coverships were washed trice with PBS followed by fixing with 4% paraformaldehyde for 20 min and then staining with DAPI to identify the nuclei for 5 min. Finally, the coverships were visualized and photographed by fluorescent microscope (Nikon, Japan).

4T1 cells were plated in 6-cell plates at a density of 1 × 10^5^ cells per cell and cultured for 24 h. Cells were treated with DOX, DOX-DGL-PEG, Angio-DOX-DGL-PEG, DOX-DGL-GNP and Angio-DOX-DGL-GNP at a final DOX concentration of 12.5 μg/mL. After incubation for 2 h, the cells were washed three times with cold PBS, trypsinized and resuspended in 0.5 mL of PBS. Then the fluorescent intensity was tested by flow cytometry (BD, USA).

### Cellular uptake mechanism study

In order to study the uptake mechanism of Angio-DOX-DGL-GNP, 4T1 cells were pre-incubated with various endocytosis inhibitors including poly-lysine (400 μg/mL), sodium azide (0.651 mg/mL), chlorpromazine (10 μg/mL), filipin (5 μg/mL) meanwhile the inhibition of free angiopep-2 (250 μg/mL) for 30 min and the effect of temperature (4°C) was also studied. Then the inhibitors were withdrawn from the wells and the cells were treated with Angio-DOX-DGL-GNP. After 1 h incubation, the solution was discarded and the cells were washed with ice-cold PBS twice and responded in 0.4 mL PBS. The fluorescence intensity was determined by flow cytometer (Cytomics™ FC 500, Beckman Coulter, Miami, FL, USA).

### Cell apoptosis assay

The apoptosis of 4T1 cells was determined by using Annexin V-FITC apoptosis detection kit (Doshido, Japan). Briefly, the 4T1 cells were plated in 6-well plates at a density of 1 × 10^5^ cells per cell and cultured for 24 h. Then the cells were treated with different formulations at DOX concentration of 12.5 μg/mL. After incubated at 37°C for 24 h, the cells were harvested, washed by PBS twice and suspended in 500 μL of binding buffer. 5 μL of Annexin V-FITC and 5 μL of propidium iodide (PI) were added into the cell suspension for 15 min incubation, respectively. The cells were immediately analysed by flow cytometry (BD, USA).

### *In vivo* imaging and tumor distribution

Animals were performed in accordance with national regulations and approved by the Institutional Animal Care and Use Committee of Sichuan University. 4T1 tumor-bearing mice were randomly into 5 groups and intravenously administrated with different formulations at a dose of 5 mg/kg DOX (equal) per mouse via the tail vein. 24 hours after administration, the mice were sacrificed and the major organs were further visualized. Images were taken on Bio-Real Quick view 3000 (Geneway International, Australia) and the fluorescence intensities were analyzed by Living Image Software.

After dehydration with sucrose solution, the tumors were embedded and frozen in OCT embedding medium (Leica, Germany), frozen slices of 16 μm thickness were prepared with cryotome Cryostat (Leica, Germany). Subsequently, the slices were stained with rabbit mAb to LRP1 antibody (1:100) overnight, followed by staining with secondary antibody Cy3-conjugated Affinipure Goat Anti-Rabbit IgG(H+L) and 0.5 μg/mL of DAPI at room temperature. After washing with PBS trice, the slices were immediately examined by a confocal microscope at corresponding excitation wavelength (LSM710, Carl Zeiss, Germany). At the same time, the slices were stained with Rab mAb to CD34 antibody (1:100) and Alexa Fluor 594-conjugated Affinipure Donkey Anti-Rabbit IgG secondary antibody with a procedure established previously. Then the distribution was determined by a confocal microscope above at corresponding excitation wavelength.

### *In vivo* therapy studies

The therapy studies were conducted in 4T1 tumor models. Briefly, 4T1 cells (5 × 10^5^) were injected into the right flank of BALB/c mice (20 ± 2). BALB/c mice bearing 4T1 tumor were randomly divided into six groups (*n* = 6) and treated with different formulations at a dose of 5 mg/kg of DOX and saline via intravenously injection at an interval of 2 days for 4 times, respectively. The tumor size and body weight of mice were recorded at the meantime and the tumor volume was calculated using the formula: Tumor volume = (width^2^/length)/2. At the end of the experiment, all the mice were killed and the main organs were isolated. Finally the tumors were imaged and weighed. For HE staining, formalin-fixed tissues were embedded in paraffin blocks and observed by optical microscope (Axiovert 40 CFL, Carl Zeiss, Germany).

### Statistical analysis

All data were displayed as mean ± SD. Statistical difference between two groups were performed by Students *t*-test. *P* value < 0.05 and < 0.01 were considered indications of statistical difference and statistically significant difference respectively.

## CONCLUSIONS

In summary, Angio-DOX-DGL-GNP was designed to overcome the limitations of current NPs such as low targeting efficiency and poor penetration ability. Based on the enhanced accumulation of angiopep-2 mediated active targeting and size-reduction of enhanced penetration efficacy, Angio-DOX-DGL-GNP significantly inhibited tumor growth and provided a promising nanoplatform for TNBC treatment.

## SUPPLEMENTARY DATA FIGURES


