# Supplementary material for: Peptide mediated active targeting and intelligent particle size reduction-mediated enhanced penetrating of fabricated nanoparticles for triple-negative breast cancer treatment
Source: Oncotarget. 2015 Oct 12;6(38):41258–74. doi: 10.18632/oncotarget.5692 (PMC4747404; doi:10.18632/oncotarget.5692)
Supplement: Supplementary file 1 [file oncotarget-06-41258-s001.pdf]

SUPPLEMENTARY DATA

In vitro cytotoxicity assays

The *in vitro* anti-proliferation activity of various materials was evaluated by the MTT assay. Briefly, HUVEC cells were seeded at a density of 3×10^3 cells/well into 96-well plates, after 12 h incubation at 37 °C in 5% CO_2 atmosphere, these cells were treated with a series of different concentration of DGL, DGL-PEG and DGL-GNP for 48 h. To assess cell viability, MTT (5 mg/mL, 20 μL) was added into each well and incubated at 37 °C for 4 h. The medium was removed and 150 μL of DMSO was added to each well at 75 rpm for 15 min. After that the percentage of cell viability was detected at 570 nm by fluorescence spectrophotometer.

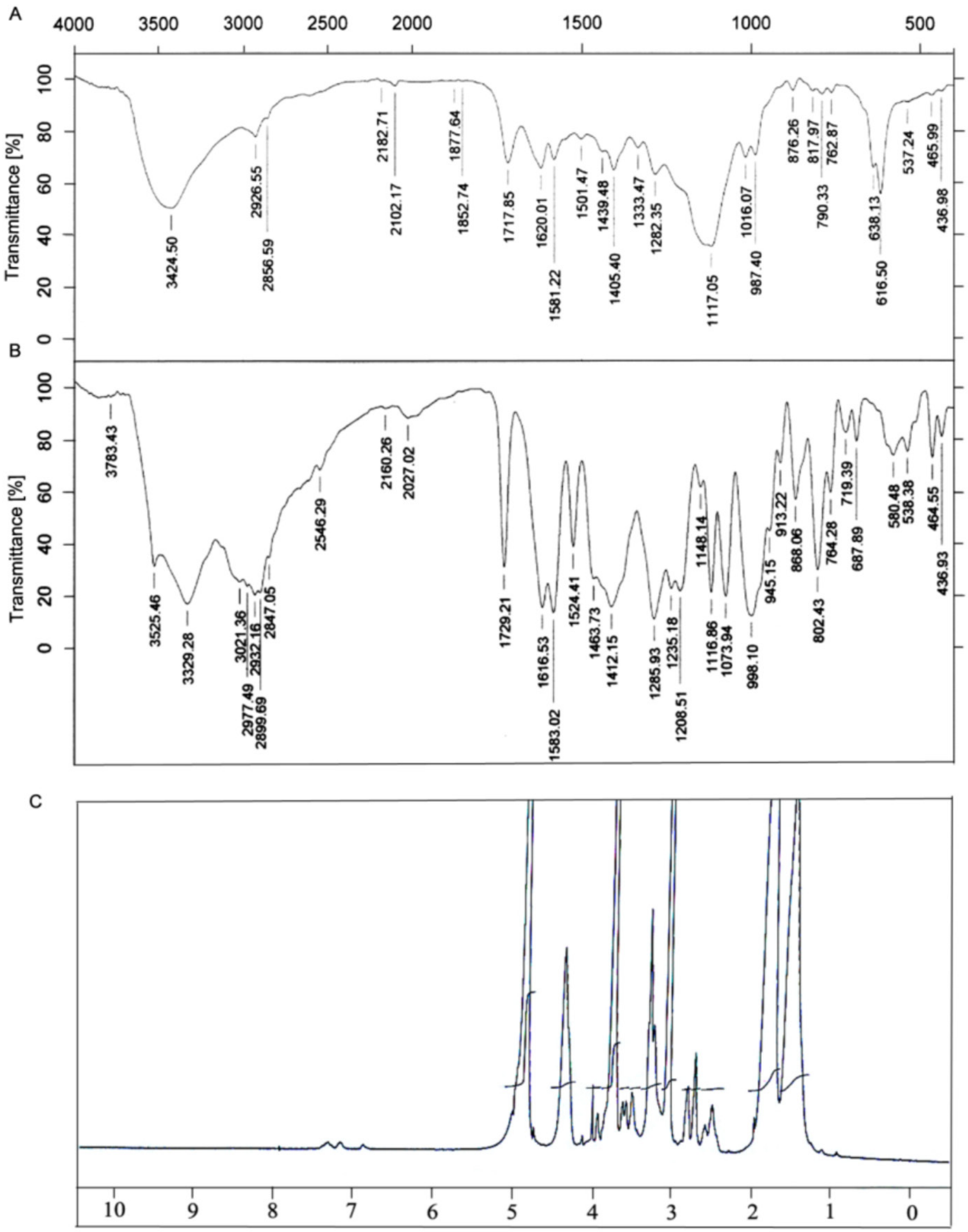

Supplementary Figure S1: FT-IR spectra of CAD A. and DOX **B.** H-NMR spectra of Angio-DGL-PEG C.

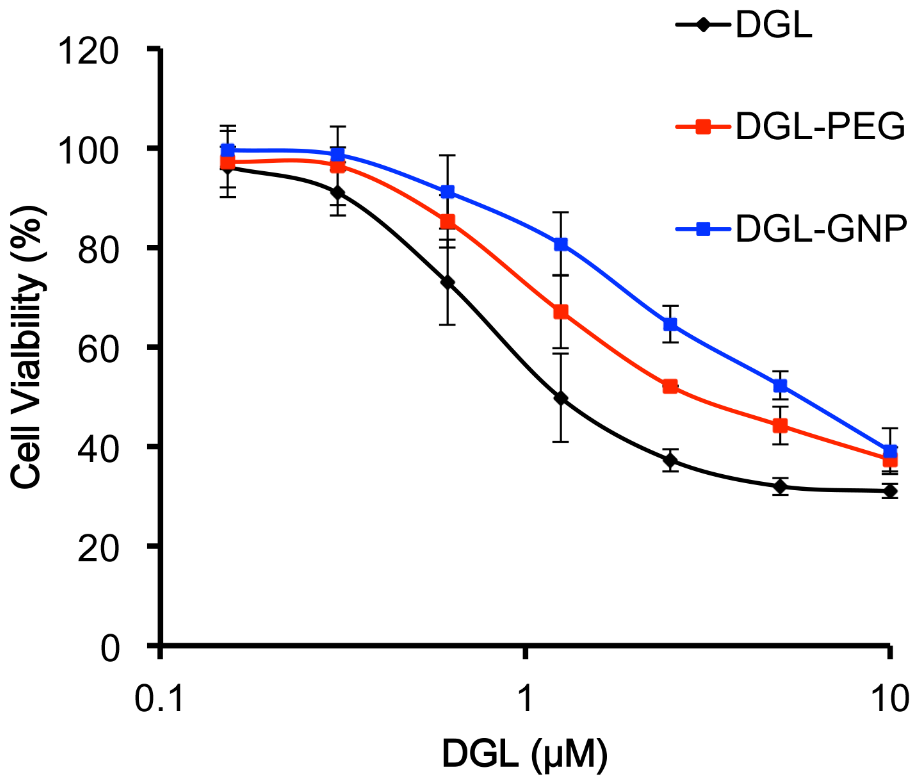

Supplementary Figure S2: The cell viability of HUVEC incubated with DGL, DGL-PEG and DGL-GNP.

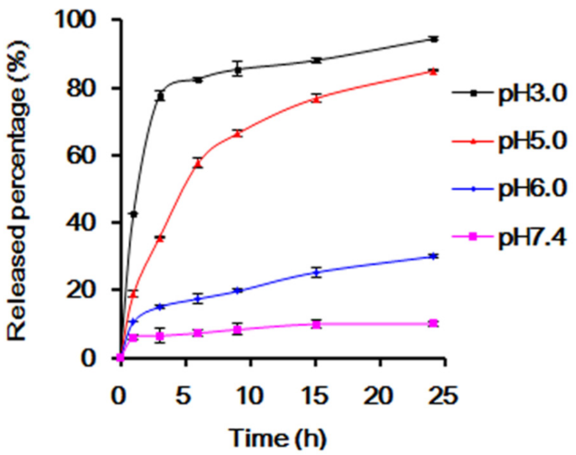

Supplementary Figure S3: ***In vitro*** **release profiles of Angio-DOX-DGL-GNP at pH 7.4, 6.0, 5.0 and 3.0.**

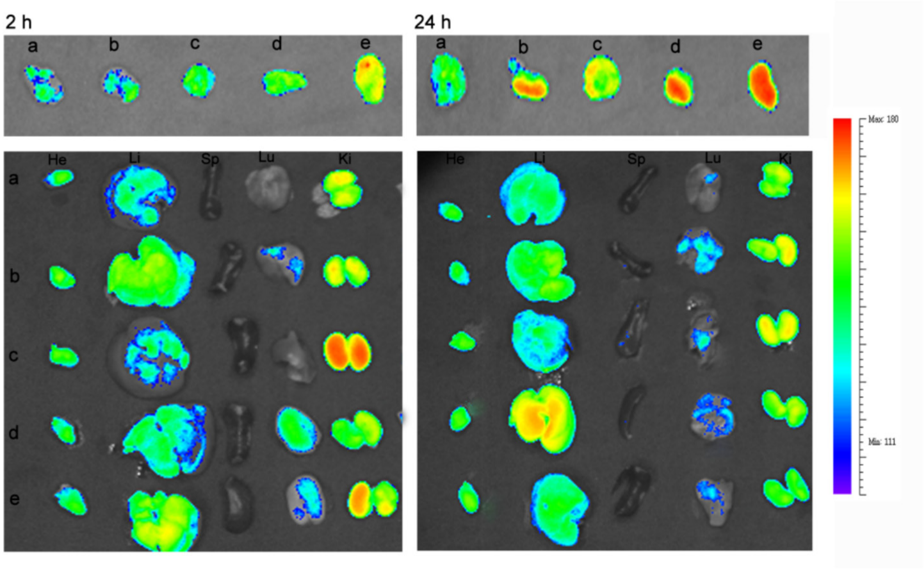

Supplementary Figure S4: *Ex vivo* fluorescence imaging of the tumor and normal tissues of 4T1 tumor-bearing BALB/C mice after 2 h or 24 h post-injection of different DOX formulations (a: DOX, b: Angiopep-DOX-DGL-PEG, c: DOX-GNP, d: DOX-DGL-GNP, e: Angiopep-DOX-DGL-GNP; He: Heart, Li: Liver, Sp: Spleen, Lu: Lung, Ki: Kidney, Tu: Tumor).
